# In vitro effects of two silicate-based materials, Biodentine and BioRoot RCS, on dental pulp stem cells in models of reactionary and reparative dentinogenesis

**DOI:** 10.1371/journal.pone.0190014

**Published:** 2018-01-25

**Authors:** Ludwig Stanislas Loison-Robert, Mathilde Tassin, Eric Bonte, Tsouria Berbar, Juliane Isaac, Ariane Berdal, Stéphane Simon, Benjamin P. J. Fournier

**Affiliations:** 1 School of Dentistry, Paris Descartes University, Sorbonne Paris Cité, Paris, France; 2 « Molecular Oral Pathophysiology » group, INSERM UMRS 1138, Cordeliers Research Center, Paris Descartes University, Paris, France; 3 « Molecular Oral Pathophysiology » group, INSERM UMRS 1138, Cordeliers Research Center, Pierre and Marie Curie University, Paris, France; 4 UMRS 1138, Cordeliers Research Center, Paris Diderot University, Paris, France; 5 Faculty of Dentistry, Paris Diderot University, Sorbonne Paris Cité, Paris, France; Università degli Studi della Campania "Luigi Vanvitelli", ITALY

## Abstract

**Background:**

Calcium silicate-based cements are biomaterials with calcium oxide and carbonate filler additives. Their properties are close to those of dentin, making them useful in restorative dentistry and endodontics. The aim of this study was to evaluate the *in vitro* biological effects of two such calcium silicate cements, Biodentine (BD) and Bioroot (BR), on dental stem cells in both direct and indirect contact models. The two models used aimed to mimic reparative dentin formation (direct contact) and reactionary dentin formation (indirect contact). An original aspect of this study is the use of an interposed thin agarose gel layer to assess the effects of diffusible components from the materials.

**Results:**

The two biomaterials were compared and did not modify dental pulp stem cell (DPSC) proliferation. BD and BR showed no significant cytotoxicity, although some cell death occurred in direct contact. No apoptosis or inflammation induction was detected. A striking increase of mineralization induction was observed in the presence of BD and BR, and this effect was greater in direct contact. Surprisingly, biomineralization occurred even in the absence of mineralization medium. This differentiation was accompanied by expression of odontoblast-associated genes. Exposure by indirect contact did not stimulate the induction to such a level.

**Conclusion:**

These two biomaterials both seem to be bioactive and biocompatible, preserving DPSC proliferation, migration and adhesion. The observed strong mineralization induction through direct contact highlights the potential of these biomaterials for clinical application in dentin-pulp complex regeneration.

## Introduction

Dentistry (restorative, endodontics or prosthodontics) aims to conserve and protect tooth and jaw bone integrity. An important means to achieve this is to prevent exposure of the dentin-pulp complex to the microenvironment of the oral cavity. Schematically, the tooth is composed of two protective layers of mineralized hard tissues, enamel and dentin, encapsulating a loose connective tissue, the pulp. Keeping the pulp vital is a priority to ensure the long-term functionality of the tooth. Thus, once the pulp is exposed to the oral cavity, a sealing is required.

Cells of the dental pulp are at risk of cell death brought on by a variety of circumstances such as dental caries, trauma and operative dental procedures. These induce tertiary dentinogenesis needed to maintain pulp vitality [1]. Two types of dentinogenesis are well described in the literature. Reactionary dentinogenesis is the process whereby dentin is secreted in response to a local stimulus that reactivates the resting odontoblasts [2]. Reparative dentinogenesis occurs when odontoblast cells die, thus initiating a complex regenerative process that allows the formation of reparative dentin following recruitment of progenitor cells and their differentiation into odontoblast-like cells [1,3]. Human dental pulp stem cells (DPSC) share the same embryologic origin as odontoblasts, are able to self-renew and differentiate toward several lineages [4]. One of the most striking features of DPSC for dental tissue engineering applications is their odontogenic potential [3]. DPSC play an important role in the healing process through their ability to undergo odontoblast-like cell differentiation. Thus, DPSC provide an excellent model for studying the *in vitro* biological effects of biomaterials on tertiary dentinogenesis.

Engineered biomaterials should be biocompatible, bioactive and able to fill and restore a tooth. The use of hydraulic cements [5] in dentistry has become a method of choice to protect the dentin-pulp complex. Tricalcium silicate-based cements such as Biodentine^TM^ (BD) (Septodont, Saint Maur des fossés, France) have recently been commercialized and have a wide range of applications, including in pulpotomy, pulp capping and endodontic repair (root perforations, apexification, resorptive lesions, and retrograde filling in endodontic surgery) [6]. A new hydraulic cement, BioRoot RCS (BR), was recently marketed as a mineral root canal sealer. To date, only two studies have investigated this material *in vitro* [7,8], but neither compared it with BD nor studied its effects on DPSC. The use of BD in direct pulp contact showed complete dentinal bridge formation and absence of an inflammatory pulp response. Also, Zanini et al. evaluated the biological effect of BD on a murine pulp cell line (OD-21) by analyzing the expression of several biomolecular markers after culturing OD-21 cells with or without BD [9]. Their results, consistent with other studies, demonstrated that BD is bioactive due to its ability to stimulate OD-21 cell proliferation and biomineralization [10]. BD may be used as a dentin substitute in several clinical indications. A study of its interactions with pulp cells demonstrated its biocompatibility and its ability to induce odontoblast differentiation and mineralization in cultured pulp cells [11]. In contrast, however, the effect of BR on the differentiation of pulp cells to odontoblast-like cells has not been fully investigated.

As the effect of BR on human DPSC has never been examined, the purpose of this study was twofold: 1) to evaluate the *in vitro* response of DPSC—in terms of their morphology, proliferation, migration, adhesion, biocompatibility and molecules secreted—to contact with this new hydraulic cement; and 2) to investigate mineralization induction. Moreover, both BD and BR must be placed in direct contact with the periodontium or with pulp tissue to promote reparative dentinogenesis. An original facet of this study is its use of a thin agarose membrane interposed between the cells and the hydraulic cements to assess the effects of diffusible molecules released by the biomaterials in a process mimicking reactionary dentinogenesis. No previous studies have investigated the effects of such indirect contact of BD and BR through a membrane, as undertaken for some other materials [12]. Thus, the effectiveness of the two materials when placed in direct contact with DPSC versus when partitioned by a thin gel layer was studied to interrogate the role of soluble factors on cell fate.

## Materials & methods

### Cell isolation and culture

Stem cells were extracted from human dental pulp tissue. Patients had no systemic pathology, oral infection or disease. All patients gave their informed consent according to the Helsinki Declaration (1975), this was approved by local ethics committee (Département de la Recherche Clinique et du Développement-IDRCB n°2015-A01509-40). Pulp tissue was obtained following extraction as already described (Gronthos et al., 2000). Cells were seeded and routinely maintained in low-glucose Dulbecco’s modified Eagle’s medium [DMEM 1X-GlutaMAX™ (Gibco®)] containing 10% heat-inactivated Fetal Bovine Serum (FBS), 1% non-essential amino acids MEM 100X (Gibco®), 1% Penicillin-Streptomycin (10,000 U/mL) (Gibco®) and 0.5% Fungizone® Antimycotic (Gibco®) and 50 μg/mL 2-phospho-L-ascorbic acid trisodium salt (Sigma-Aldrich) at 37°C in a humidified 5% CO2 incubator.

### Preparation of silicate cement solutions

Biodentine^TM^ and BioRoot RCS were prepared according to the manufacturers’ instructions under aseptic conditions and transformed into powder by grinding under cold conditions (-20°C) and then sterilized by dry heat.

### Basal medium with no red phenol

The basal medium for the two biomaterials was DMEM (without phenol red) containing 10% FBS, 1% non-essential amino acids MEM 100X (Gibco®), 1% Penicillin-Streptomycin (10,000 U/mL) (Gibco®), 0.5% Fungizone® (Gibco®) and 50 μg/mL 2-phospho-L-ascorbic acid trisodium salt (Sigma-Aldrich).

### Biomaterial stock solutions

The ground powder was added to the basal medium to prepare stock solutions (10 mg/mL) of each material and vortexed. For each calcium silicate cement, a final concentration of 2 mg/mL was used.

### Scanning electron microscopy

First, samples of calcium silicate cement powder taken before and after preparation (see above) were placed on coverslips, fixed and examined by SEM. In addition, DPSC were seeded on 0.2% gelatin coated-coverslips at a density of 30×10^3^ cells per well in a 24-well plates for 7 days. The cells were allowed to adhere for 2 h in 800 μL of basal solution. Then, 200 μL of biomaterial stock solution was added and the basal medium was changed every 3 days. The coverslips were fixed with 4% paraformaldehyde/5% sucrose solution, then with 2.5% glutaraldehyde. After rinsing, the coverslips were incubated with 0.5% osmium tetroxide for 60 min, dehydrated in specific grade alcohol, attached to sample holders, and then coated with gold. The samples were examined by SEM. Microphotographs were taken at magnifications of ×400, ×1000, ×2000 and ×5000.

### Indirect contact model

To design an *in vitro* model mimicking reactionary dentinogenesis, we spread the cells and separated them from each of the two calcium silicate materials by using a thin layer of 2% agarose prepared from a low-temperature gelling solution.

### Direct and indirect cell contact proliferation assays

The rate of proliferation was estimated by performing MTT analysis on days 0, 1, 5 and 8 (Life Sciences).

Cell proliferation was also investigated using antibody Ki67 and nuclear DAPI staining (see below). Proliferation indexes were assessed by determining the total number of cells stained positively for Ki67 divided by the total number of DAPI-stained nuclei.

### FACS analysis

Cells were detached using 0.02% Trypsin/EDTA solution in phosphate-buffered saline (PBS), collected by centrifugation (5 min at 300g), washed in 3% FBS in PBS at 4°C, then incubated in solutions of Annexin V and 7-AAD in 3% BSA in PBS for 60 min at room temperature. Cells were then washed 3 times by centrifugation at 400 g for 5 min and suspended in 3% FBS.

### Cell viability assay

Cells were cultured for 24 h with each of the two biomaterials and for 30 min in the presence of CellTraceCalcein Red-Orange AM (Life Technologies, Carlsbad, CA, USA) as indicated by the supplier. Samples were observed and digital images recorded using a Zeiss microscope equipped with a digital camera.

### pH measurements

Conditioned media from the proliferation assay were set aside and then samples were collected after 24 h, 5 days, and 8 days for pH measurement using a pH meter (Hanna Instruments, pH 210). The electrode was inserted into the soaking solutions at room temperature (24°C) and each measurement was repeated three times. The mean pH was then plotted with recording time.

### Direct and indirect cell contact cytotoxicity assays and genotoxicity

For cell cytotoxicity assays, cells were studied at confluency. The medium was removed at day 0. For direct contact, 800 μL of basal solution and 200 μL of material stock solution were directly added together. For indirect contact, 200 μL of 2% agarose low-gelling temperature solution was placed on the cells for 1 h at 37°C to jellify. Then, 800 μL of basal solution and 200 μL of material stock solution were added. 1 mL of basal solution without biomaterial served as a control. Cell viability was estimated by performing the MTT assay on days 0, 1, 5 and 8, as described by the supplier (Life Sciences).

Cell apoptosis and death were evaluated by using a Biotium Kit (Annexin V–Propidium Iodide) as indicated by the supplier.

For cell genotoxicity assays, cells were studied after 7 days in contact with the materials. Cells on coated coverslips were fixed in PBS (pH 7.4) containing 4% paraformaldehyde / 5% sucrose for 10 min, rinsed three times in PBS, and permeabilized using 0.25% Triton X-100 for 15 min at room temperature (RT). To block nonspecific staining, cells were treated with PBS containing 1% BSA/1% glycine at 37°C for 30 min. Samples were then incubated with 1/1000 pH2AX antibody at 37°C for 1 h. Samples were then incubated with Alexa Fluor® 555 goat anti-mouse IgG (H+L) at 37°C for 30 min. Cell nuclei were stained using Hoechst 33342.

### Wound healing assay

Approximately 30×10^3^ cells per well in triplicate in a 24-well plate were seeded. When a complete confluence monolayer was obtained, the cells were incubated for 24 h in medium without FBS to synchronize the cells. Then a scratch was made through the confluent cell monolayer with a 200-ml pipette tip. After wounding, the cells were washed with PBS to remove cellular debris and then incubated by direct contact with 800 μL of basal solution and 200 μL of material stock solution for 24 h, 48 h or 7 days. 1 mL of basal solution served as a control. Microphotographs were taken after scratching. The cultured cells were observed with a Zeiss microscope equipped with a digital camera. Scratch-wound closure rate was determined using ImageJ software: we measured the distance from the origin of the wound to the point where each of the cells had migrated.

### Immunohistochemistry

After 7 days in contact with the materials, coated coverslip cultures were fixed in PBS (pH 7.4) containing 4% paraformaldehyde/5% sucrose for 10 min. For detection of intracellular molecules, the cells on the coverslips were permeabilized using 0.5% Triton X-100. To block background staining, cells were treated with PBS containing 1% BSA/1% glycine at 37°C for 20 min. Samples were incubated with the primary antibody at 4°C overnight or at 37°C for 2 h. For double immunostaining, primary antibodies were incubated as above. Samples were then incubated with the appropriate secondary antibodies at 37°C for 1 h. Cell nuclei were stained using DAPI.

The antibodies used for immunostaining were: 1/100 monoclonal anti-fibronectin (Sigma-Aldrich) and HFN 7.1 (which was deposited into the DSHB by Klebe, R.J. (DSHB Hybridoma Product HFN 7.1)), 1/150 anti-collagen I (ab292; Abcam), anti-Ki67 (ab15580; Abcam), 1/50 monoclonal anti-vimentin (3CB2 was deposited into the DSHB by De La Rosa, E.J. (DSHB Hybridoma Product 3CB2)), 1/50 rat monoclonal anti-tubulin (Abcam, ab6160). Secondary antibodies were Alexafluor 488-conjugated goat anti-rabbit, Alexafluor 594-conjugated goat anti-mouse, Alexafluor 594-conjugated goat anti-rat, Alexafluor 546-conjugated goat anti-mouse and Alexafluor 488-conjugated goat anti-mouse (Life Technologies Corporation). Samples were observed using a Zeiss microscope equipped with a digital camera.

### In vitro mineralization assay

For the induction of mineralization, DPSC cultures were seeded in 24-well plates at a density of 30×10^3^ cells per well and in 6-well plates for RNA at a density of 100×10^3^ cells per well. At 80% confluency, DPSC were induced for differentiation assays. Three types of media were used, with or without the two materials, in direct or indirect contact (Table 1). The cells were cultured for a total of 10 days and the differentiation media were changed every 3–4 days.

**Table 1:** Media used in the study.

	Composition
*Classic medium*	DMEM low glucose + Antibiotics + 10% FBS + 1x AANE + 50 μg/mL L2 phosphate ascorbic acid
*Medium 1*	Classic medium + 10 mM betaglycerophosphate
*Medium 2*	Medium 1 + 10 nM Dexamethasone

For the assessment of *in vitro* mineralization, cells were washed twice with PBS and fixed in PBS containing 4% paraformaldehyde/5% sucrose for 15 min at 4°C. Then, the cells were washed with deionized H2O (dH2O) and stained with 1% AR-S (Sigma–Aldrich) (pH 4.2) for 30 min at RT. Cells were then rinsed three times with dH2O to reduce nonspecific staining. Mineralized nodules were visualized and photographed using a Zeiss microscope equipped with a digital camera. Staining was quantified by using the method of Gregory et al. (Gregory, Gunn, Peister, & Prockop, 2004). Experiments were performed in triplicate wells and repeated at least three times.

### Analysis of mRNA expression by qRT-PCR

RNA was extracted from cultured cells using Tri Reagent solution (Molecular Research Center) according to the manufacturer's protocol. RNA concentration and purity were evaluated by spectrophotometry (Nanodrop, Thermo Scientific, Wilmington, MA, USA). Total RNA concentration and purity were measured and samples with an OD260/280 ratio above 1.8 were used for the study. cDNA was synthesized using the Superscript II kit (Invitrogen, France) according to the manufacturer’s instructions. All primers were designed to anneal to the boundaries of exons when possible, and analyzed by BLASTn software (http://blast.ncbi.nlm.nih.gov/Blast.cgi) for specificity and DNA-fold for secondary structures. For the qRT-PCRs, diluted reverse-transcribed products were mixed with 7.5 μL of 2X Kapa SYBR Fast qPCR (KapaBiosystems, Wilmington, MA, USA) mix and 5 pmoles of primers, for a final volume of 15 μL. Real-time PCR amplification was performed on a CFX96 System (Bio-Rad Laboratories) using the following program: one cycle at 94°C for 3 min, 35 cycles at 95°C for 5 s, gene-specific annealing for 20 s, and reaction completion with reading plate and a melt curve analysis from 65°C to 95°C, 5 s for each 0.5°C. Amplification reactions were conducted for target genes, with succinate dehydrogenase complex subunit A flavoprotein (SDHA) and ubiquitin C (UBC) as the reference genes. Non-transcribed RNA samples and a water control were used as negative controls. The PCRs were performed in triplicate for each sample. Gene expression analysis was performed using three parallel cell lines. The data were analyzed based on the comparative 2ΔΔCt method (CFX Manager Software Version 2.1, Bio-Rad Laboratories). For primer sequences, see S2 Table. In a set of experiments, the PCR products were also run on agarose gels (1.5%) to confirm the correct amplicon size.

### Statistical analysis

Statistical analysis was performed using two-way ANOVA (Dunn test) and was carried out using Prism GraphPad 5 software (GraphPad Software Inc, La Jolla, CA, USA). Results are presented as mean ± standard deviation (SD). Results were considered significant with a *p* value <0.05. (on graphs *: p<0.05; **: p<0.01; ***: p<0.001; ****: p< 0.0001).

## Results

### Examination of cell morphology and biomaterials

**Fig 1:**
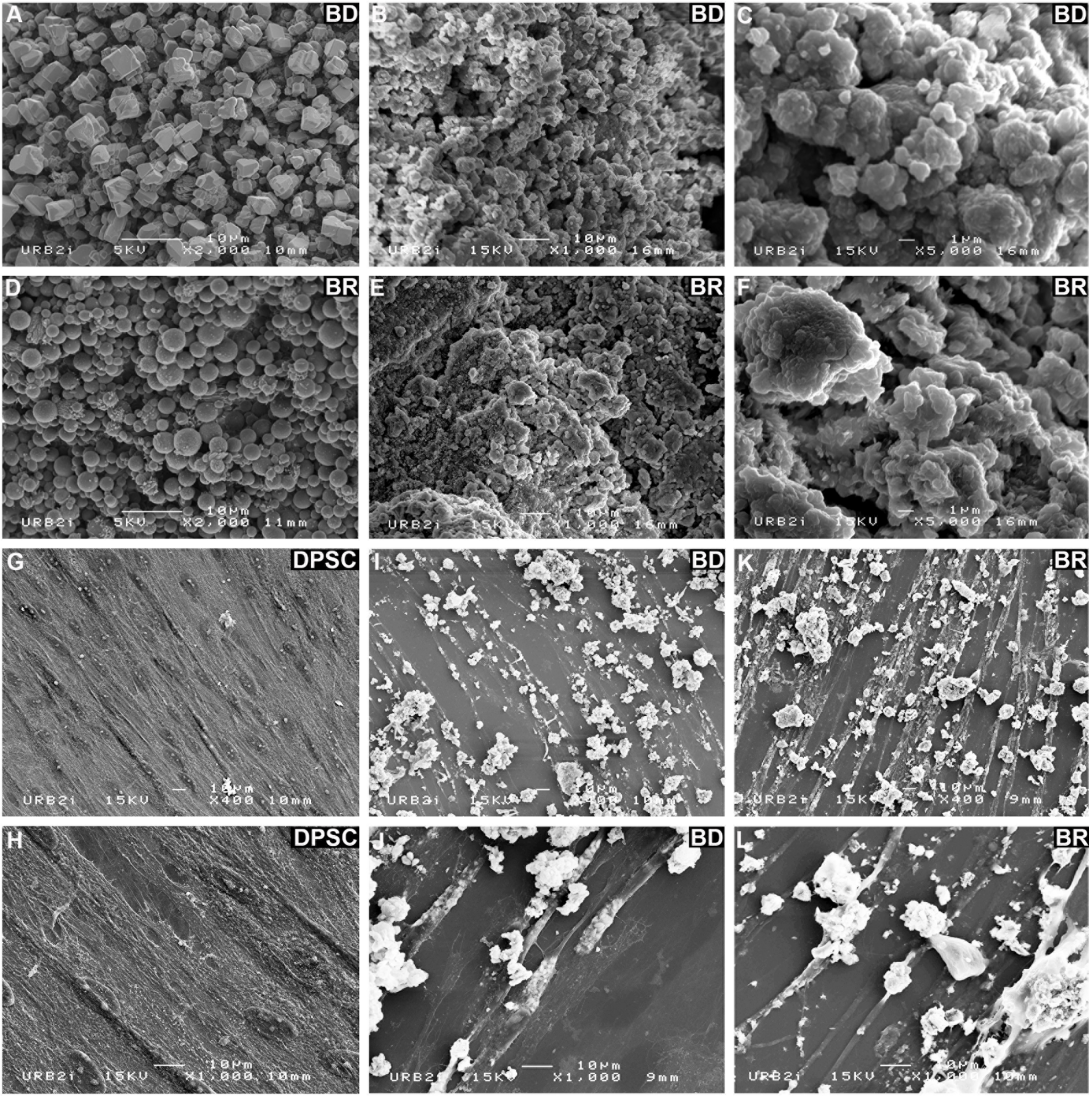
SEM images of BD and BR before (panels A and D) and after mixing (panels B, C, E and F). SEM observations at different magnifications (×400, G, I, K and ×1000, H, J, L) of DPSC alone (G and H), in contact with BD (I and J), and in contact with BR (K and L).

According to the manufacturers’ literature, BD and BR are of broadly similar chemical composition. However, they appeared different after preparation: BD had a yellow color whereas BR was white. In addition, BR had a fluid consistency and its setting time was longer. SEM microphotographs showed different shapes of the powder particles: BD seemed to be cuboidal whereas BR was spherical (Fig 1A and 1D). After preparation, granules and small crystals appeared on the surface of the particles (Fig 1B, 1C, 1E and 1F). SEM examination after 7 days of cell culture revealed morphologic changes in the particles as well as their affinity for cells, but no differences between the two silicate surfaces were observed. DPSC cultured with the BD and BR seemed more elongated and narrow (Fig 1I–1L) compared to pulp stem cells cultured without the biomaterials (Fig 1G and 1H). The adhesion of the biomaterial to cells was difficult to assess because the material was neither regular nor planar; however, intimate contact was observed between DPSC and both types of particulate powder (BD and BR), and some cells appeared to have particles attached to their surfaces (Fig 1G and 1L).

### Growth characteristics

**Fig 2:**
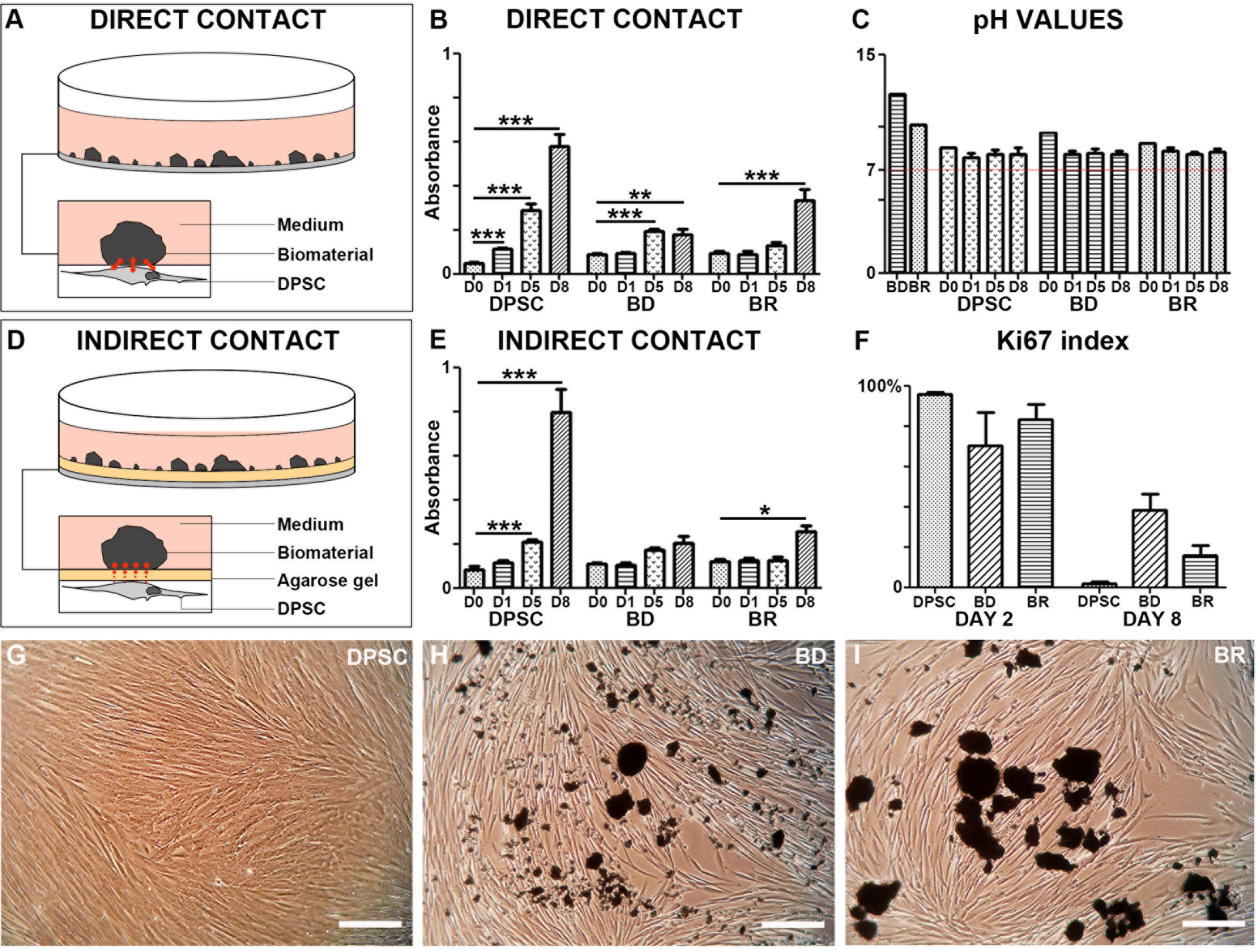
Schematic view of the direct (A) and indirect (D) experiments. Proliferation kinetics were followed using MTT assay in direct contact experiments (B) and indirect experiments (E). pH values were recorded (C) while Ki-67 indexes were calculated after immunostainings (F). Images were obtained by inverted phase contrast microscopy at day 8 of culture of DPSC alone (G), in contact with BD (H) or with BR (I). Scale bar: 100μm.

To investigate the influence of physical contact, we developed an *in vitro* model of indirect contact between cells and the biomaterials by separating the two with a thin layer of 2% agarose gel (Fig 2A and 2D). We then used a proliferation assay to compare the growth of cells in direct versus indirect contact with each of the two biomaterials. Differences in growth rates of DPSC cultures with versus without the materials were observed. In direct contact with BD or BR, the cells proliferated more slowly (and needed more days to reach confluency) than cells cultured without biomaterials. All cultures, whether in direct or indirect contact with the biomaterials, showed similar patterns of cell proliferation, with the greatest proliferation obtained at day 8 compared to day 0 (except BD in indirect contact) (Fig 2B and 2E). At day 8, the Ki-67 indexes for the BD and BR cultures were higher than that of the control culture, confirming that the cells were not confluent but still proliferating (Fig 2F). Cytological examination by phase contrast microscopy revealed that the cells in direct contact with the biomaterials were indeed non-confluent, unlike control DPSC (Fig 2G and 2I). One possible explanation for the reduced cell growth is that the biomaterials might reduce the space available for cell proliferation. However, we observed the same phenomenon in the indirect contact experiments. Therefore, we concluded that the presence of the two materials, whether in direct or indirect contact, reduced the proliferation rate of DPSC by increasing the cell doubling time.

### pH measurements

In DMEM medium with BD, the pH was initially 12.24, decreased to 8.18 (±0.8) at 24 h, and remained stable for 8 days. For DMEM medium with BR, the pH varied from 9.63 to 8.35 (±0.7) over a period of 8 days (Fig 2C). No differences between pH values were observed for the direct and indirect contact experiments (data not shown).

### Viability, apoptosis and cell death

**Fig 3:**
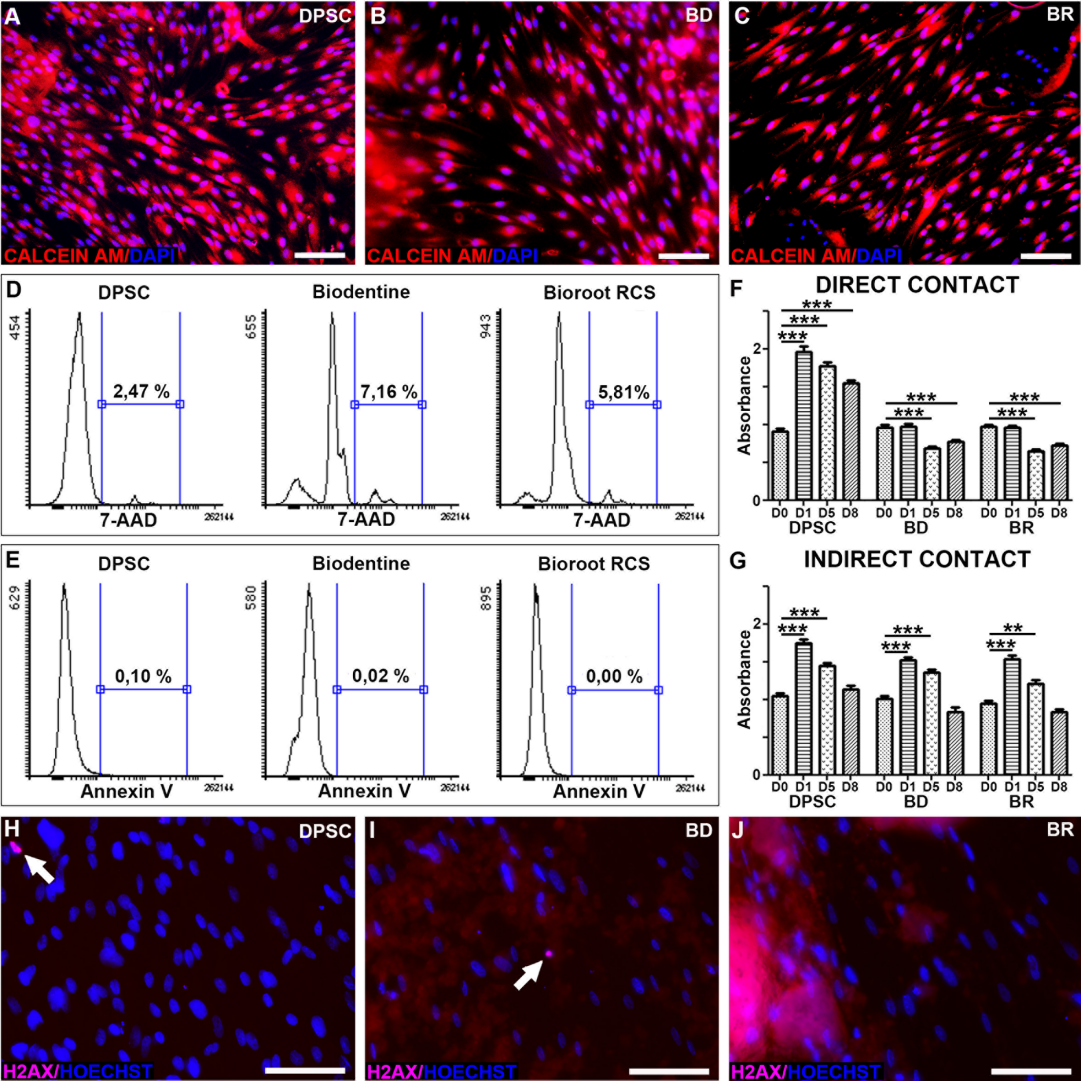
Cell viability was assessed using Calcein-AM assay on DPSC without biomaterials (A), in contact with BD (B) and in contact with BR (C). Genotoxicity was evaluated using phospho-H2AX immunostaining under the same conditions (H, I and J, respectively). Cell death was examined by using 7-AAD and flow cytometry (D), and apoptosis by using Annexin-V staining (E). Cytotoxicity was measured by MTT assay in direct contact (F) and indirect contact (G) conditions. Scale Bar: 100μm.

To assess whether the two biomaterials cause changes in cell viability or apoptosis, cells at 70% of confluency were incubated for one week in contact with BD or BR. Calcein-AM fluorescence revealed that most of the cells in contact with the biomaterials were alive (Fig 3B and 3C), similar to the control cells (Fig 3A). However, 7-amino-actinomycin D (7-AAD) staining and flow cytometric analysis showed that the number of non-viable cells was increased more than twofold in comparison to control cells without biomaterials: 7.16% for BD, 5.81% for BR, and 2.47% for the control (Fig 3D). Apoptosis was investigated by flow cytometric detection of Annexin V staining. No apoptosis was observed among cells cultured in contact with BD or BR (Fig 3E).

### Cytotoxicity and genotoxicity

To further analyze cytotoxicity, we performed MTT assays on confluent cells exposed to the biomaterials for 8 days in direct or indirect contact. Confluent DPSC, characterized by a very low proliferative rate, were used to mimic the *in vivo* condition of tissue in the absence of any cellular damage. In direct contact with either biomaterial, a progressive loss of cell number was observed at days 5 and 8 (Fig 3F). Moreover, when compared to control cells, the biomaterials induced a significant reduction in MTT absorbance at day 8, meaning that fewer cells were present (Fig 3F). To determine if direct contact or a diffusible substance(s) was involved, the same experiment was carried out, but with the cells and biomaterials in indirect contact (i.e., separated by a 2% agarose layer). Under this condition, there was no significant reduction in cell number at day 8 (Fig 3G), indicating that physical contact might cause cellular death or a reduced proliferation rate—there was no increase in cell number at day 1 of culture in the direct contact experiments. This lack of proliferation might be a consequence of reduced space for cell growth owing to the presence of biomaterial particles in the culture dishes. However, in comparing the cytotoxicity of the two biomaterials after 8 days, there was no significant difference between BD and BR either in direct (Fig 3F) or indirect (Fig 3G) contact.

Genotoxicity was assessed using H2AX phosphorylation as a marker of DNA double strands breaks. There was no significant positivity for H2AX phosphorylation in cells exposed to BD or BR (Fig 3H–3J), underlining the absence of genotoxicity of the two biomaterials.

### Migration test

**Fig 4:**
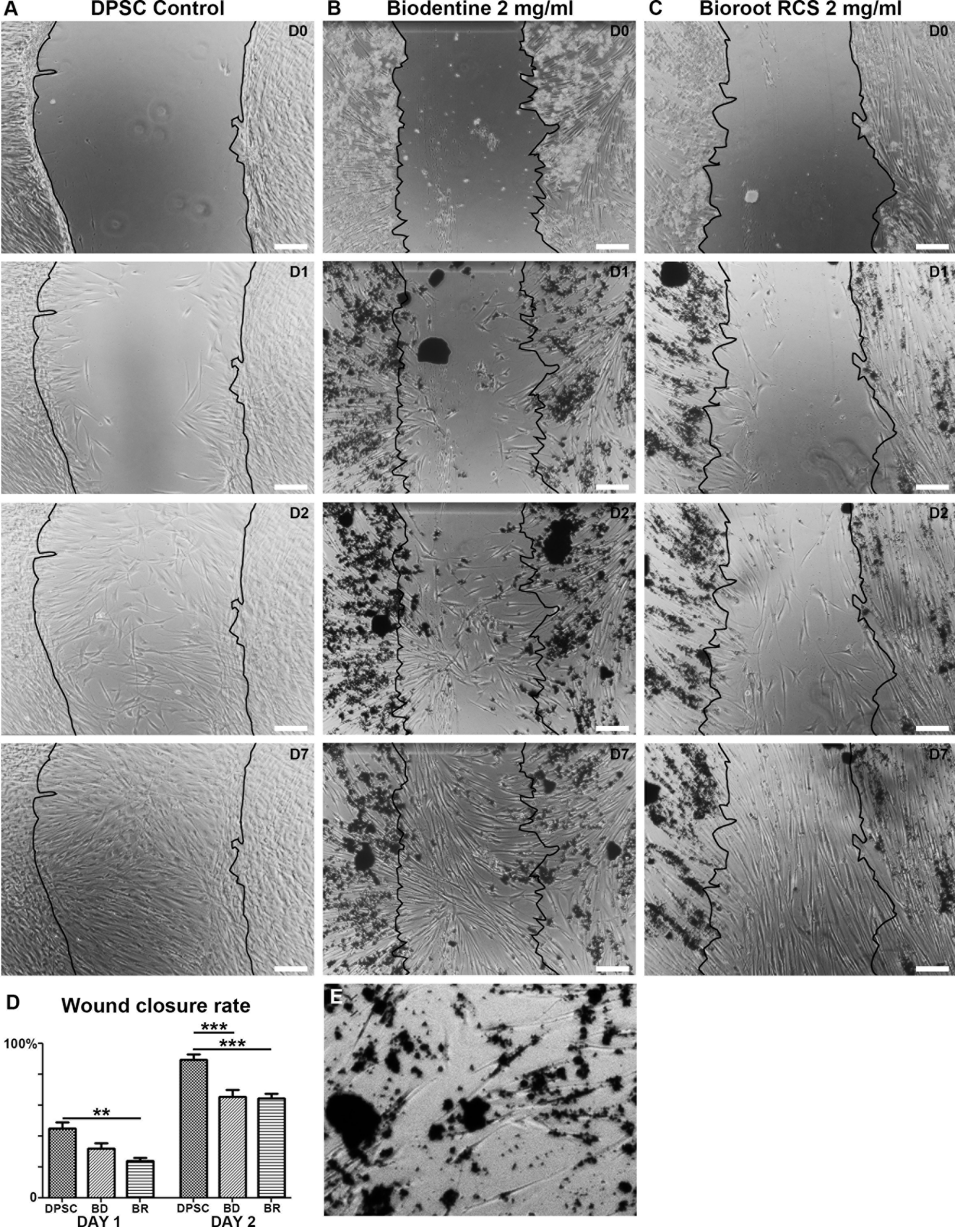
Scratch assays were performed on DPSC (A), DPSC with BD (B) and with BR (C) and followed daily by phase contrast microscopy. Wound closure rates were evaluated using image analysis software, ImageJ (D). Higher magnification showed the close contact between cells and the biomaterials (E).

To explore a potential effect of the two biomaterials on cell motility under direct contact, a wound healing assay was performed. By monitoring cell movement at days 0, 1, 2 and 7, the results showed that the two biomaterials delayed closure of the wound edges, compared with the control (Fig 4A, 4B and 4C). However, after 2 days, no significant differences were observed between BD and BR (Fig 4D). Moreover, as depicted in Fig 4E, the cells seemed to be attracted to the biomaterials (Fig 4E), partly explaining the closure delay.

### Cytoskeleton and extracellular matrix

**Fig 5:**
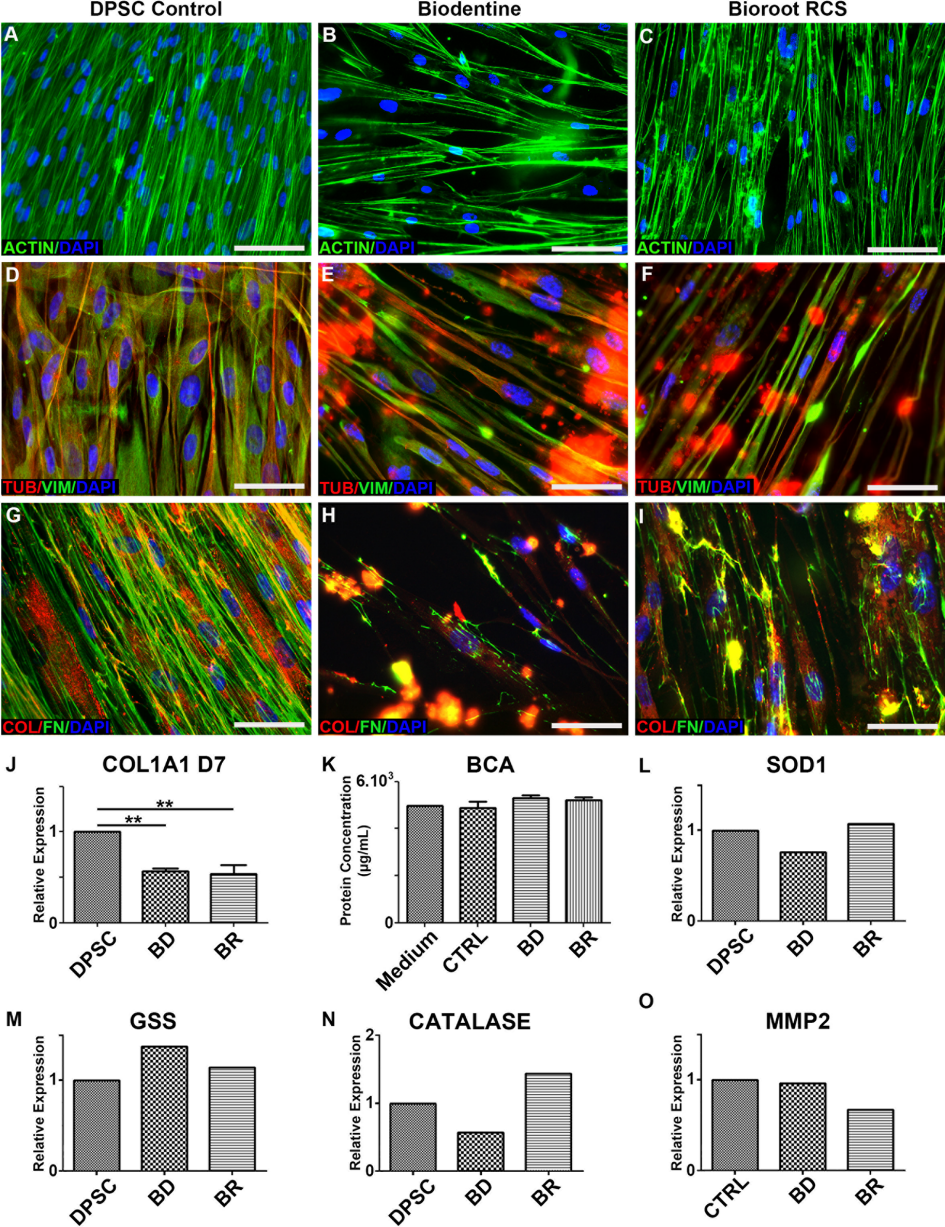
Cytoskeleton and major extracellular matrix proteins were analyzed by immunofluorescence. Actin was observed in DPSC (A), DPSC with BD (B) and in DPSC with BR (C); vimentin and tubulin (D, E and F); and type 1 collagen and fibronectin (G, H and I). The observed decrease in type 1 collagen, when DPSC were in contact with biomaterials, was confirmed using RT-qPCR (5J), whereas protein secretion was not modified by the experimental conditions (Fig 5K). Scale Bar: 100μm.

At day 7 of culture, the main components of the cytoskeleton, actin, tubulin and vimentin, were observed in direct contact with the biomaterials by immunohistochemical staining, and were not altered compared to the control (Fig 5A–5F). Thus, the biomaterials did not disrupt the cellular architecture, whose integrity is necessary for cellular basal functions. However, both type I collagen and fibronectin, the two main secreted molecules in the extracellular matrix (ECM) and consistently produced by DPSC, appeared to be altered in the presence of the two biomaterials (Fig 5G, 5H, 5I and 5J). The amount of fibronectin was more deeply decreased than that of type I collagen (Fig 5I and 5J).

### Oxidative stress and the inflammatory response

First, total protein secretion (as determined by BCA) was not modified by cellular contact with the biomaterials (Fig 5K). Cellular oxidative stress can be induced in response to harmful molecules. Thus, we studied the expression of oxidative stress-associated enzymes by RT-qPCR: superoxide dismutase (SOD1), glutathione synthase (GSS), and catalase. No significant difference was observed among any of the conditions (Fig 5L–5O). Moreover, the cells did not express IL-1β or matrix metalloproteinase 9 (MMP-9) (data not shown), and matrix metalloproteinase 2 (MMP-2) was not regulated by the biomaterials (Fig 5O), whose induction is generally associated with inflammatory responses.

### Biomineralization

**Fig 6:**
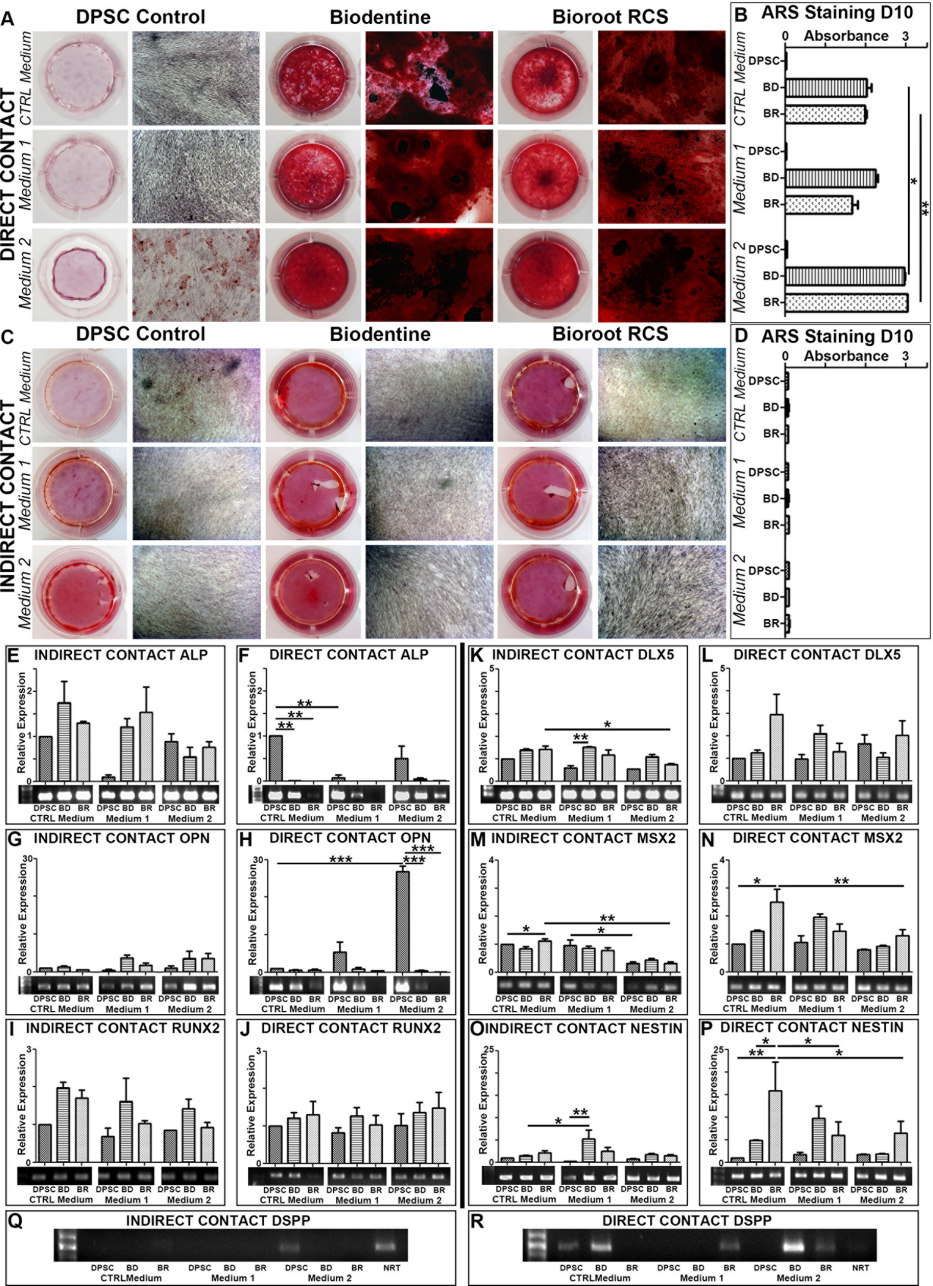
Images of Alizarin Red S staining of DPSC in the different direct contact conditions were taken using phase contrast microscopy (A), and the absorbance was measured to evaluate the degree of mineralization (B). The same experiments were repeated using indirect contact conditions (C and D). The expression of osteodifferentiation genes was quantified by RT-qPCR (E to H). The expression of odontogenic genes was analyzed (M to P). For each analysis, a representative agarose gel was run to verify the amplicon sizes. DSPP was analyzed in both direct (Q) and indirect (R) contact by conventional PCR, because we did not obtain sufficient expression to perform qPCR analysis.

One of the key characteristics of BD is its ability to form a hydroxyapatite or apatite-like layer: this phenomenon is called biomineralization. This active capacity was compared between the two biomaterials in 3 media in both direct and indirect contact experiments after 10 days. The media used were: classic medium (CTRL Medium), classic medium enriched in glycerol-phosphate (Medium 1), and osteogenic medium (Medium 2) (Table 1). Thus, to determine the mineralization effect of BD and BR on DPSC cultures, mineralized matrix deposition was examined using Alizarin Red S staining (Fig 6A and 6C). First, in direct contact with BD or BR, the cells produced mineralized matrix in all three media (Fig 6A). In contrast, in the indirect contact model, no staining, except on the edges of some wells, was observed in any of the conditions (Fig 6C). After 10 days of differentiation induction, calcium staining was quantified by spectrometry (Fig 6B and 6D). The calcium level was significantly higher in direct contact with both BD and BR in Medium 2 (Fig 6B). In CTRL Medium and Medium 1, which differ by the absence and presence supplemental organic phosphate, calcium staining was similar (Fig 6B and 6D, direct and indirect contact, respectively).

Finally, expression of target mineralization markers was studied in direct or indirect contact with cells after 10 days.

### Osteogenic markers ALP, Osteopontin, Runx2 X

ALP expression of DPSC was significantly downregulated in CTRL Medium after direct exposure to BD and BR; there was no significant difference for the other conditions (Fig 6E and 6F). Expression of Osteopontin (OPN) in DPSC was significantly increased after direct exposure to Medium 2; however, presence of the two biomaterials dramatically reduced this expression (Fig 6G and 6H). Runx2 was unmodified in all conditions compared with DPSC in CTRL Medium (Fig 6I and 6J).

### Odontogenic markers DLX5, MSX2, Nestin and DSPP

For DLX5, differences were only observed in indirect contact. DLX5 expression was increased in the presence of BD in Medium 1, compared to DPSC in Medium 1 (Fig 6K). DLX5 expression was downregulated in the presence of BR in Medium 2, compared to BR in CTRL Medium (Fig 6K). Direct contact did not reproduce these results (Fig 6L). In the case of MSX2 expression, for both indirect and direct conditions, MSX2 expression was upregulated in DPSC in the presence of BR in CTRL Medium, compared to DPSC in CTRL Medium; whereas it was reduced in DPSC in the presence of BR in Medium 2, compared to DPSC in CTRL Medium (Fig 6M and 6N). In addition, direct presence of BR increased Nestin expression in CTRL Medium and downregulated it in Medium 2 (Fig 6O and 6P). Lastly, in direct contact, DSPP expression was decreased in DPSC in Medium 2, but BD in the same medium amplified DSPP expression (Fig 6Q and 6R).

## Discussion

The use of hydraulic cements [5] in dentistry to protect the dentin-pulp complex and restore tooth integrity has become a common practice. BioRoot RCS (BR), a new calcium silicate-based cement similar in composition to Biodentine^TM^ (BD), is designed to be placed in permanent and close contact with periodontal tissue. In general, such hydraulic cements have given good results in conservative therapies. However, because BR has greater fluidity, it may be more practical in these applications. Enabling appropriate cell proliferation, migration, adhesion and mineralization is an essential quality for a biomaterial in dentin-healing applications. We thus wanted to expose pulp cells to these biomaterials and examine the cellular response. The aims of this study were: (1) to evaluate *in vitro* the effects of BR and BD on cellular responses and biomineralization capacity of human DPSC, and (2) to determine whether these biomaterials act on cells by direct or indirect contact, by interposing a thin layer of agarose gel between the biomaterials and DPSC, and to mimic reactionary dentinogenesis.

We sought to reflect the clinical conditions of direct pulp capping, in which the contact between pulp cells and biomaterials occurs indirectly through blood clots and physiologic fluids or through the dentin wall. The use of a thin gel layer may only allow for the assessment of diffusible components during reactionary dentinogenesis.

SEM examination of cell-seeded biomaterials provided visual confirmation of the interactions between the cells and the two cements after 7 days of culture. Both surface chemistry and surface topography regulate cell behavior, including cell adhesion, spreading, proliferation, and differentiation[13]. The DPSC showed a typical fibroblast shape. The presence of thin processes extending from one cell to another demonstrated there were good intercellular interactions occurring in the presence of both biomaterials.

During the aforementioned procedures, each biomaterial was in direct contact with the connective tissue and therefore had the potential to negatively affect proliferation, migration and viability of pulpal cells. We observed that cells in direct contact with the two biomaterials began to proliferate after several days, but reached confluency later than control cells without biomaterial present. A similar effect was observed in a previous study, in which proliferation of immortalized murine pulp cells was initially inhibited by BD during the first 2 days of culture, followed by an increase in proliferation [9]. In our experiments, the Ki67 index at 7 days of culture confirmed this proliferation. The non-confluence we observed might be explained by the lack of space for cell growth due to the presence of the powder [14]. Moreover, the viability of cells exposed to BD and BR showed no significant differences versus the control conditions. This diminished proliferation might be due to differentiation of the DPSC toward an odontogenic phenotype.

[15–17]The cytotoxicity of endodontic cements is of great concern because irritation of the surrounding tissue may delay periapical healing. Laurent et al. tested the cytotoxic effects of BD on human pulp cell cultures, concluding an absence of toxicity for BD [11]. We observed that direct contact with BD or BR exerted a slight cytotoxic effect, whereas the indirect contact conditions produced no evidence of cytotoxicity. The direct contact experiment was carried out to simulate certain *in vivo* conditions. As these conditions were harsher than indirect conditions, we only performed genotoxicity and apoptosis experiments in the direct conditions. None of experimental conditions revealed a genotoxic effect, as evaluated through H2AX phosphorylation [18].

Wound healing processes, which are essential for tissue regeneration, are divided into distinct phases that involve cell proliferation, adhesion and migration. A recently published article which focused on the influence of BD on human DPSC, showed that BD favorably affected healing when placed directly in contact with the pulp by enhancing the proliferation, migration, and adhesion of DPSC [19]. We did not find effects of the biomaterials on cell motility per se, but we did observe delayed closure. The cells aggregated with the biomaterials before closing the monolayer scratches, which may indicate a tropism toward these silicate cements. Culturing the cells on top of BD or BR might reveal some insights into their ability to migrate on such a substrate. Still, both biomaterials allowed healing by proliferation and migration in cell scratch assays.

Many researchers have suggested that the mechanism for stimulation of repair by deposition of mineralized tissue depends on pH and the release of various ions, including the calcium ion [20]. We found that BR and BD both increase the pH of distilled water to alkaline levels upon storage. However, when in contact with cells, the highly alkaline pH values of these biomaterial solutions return to normal, certainly due to the buffer system (carbonate buffer in DMEM), similar to what may happen *in vivo* (carbonate buffer in saliva).

Our study also investigated the effects of BD and BR on the cytoskeleton and on ECM components. The two cements did not disrupt the principal protein scaffolds of the DPSC cytoskeleton: actin microfilaments, tubulin microtubules, and the vimentin intermediate filament. However, both type I collagen and fibronectin, the two main secreted molecules in the ECM, and consistently produced by DPSC, appeared altered in the presence of the two biomaterials. *In vivo*, pulp capped with BD showed complete dentinal bridge formation and no inflammatory pulp response [21]. Expression of the molecules IL-1β and MMP-9, generally associated with inflammation of connective tissue, was not induced by either biomaterial. No oxidative stress due to the biomaterials was observed.

When the dentin barrier is disrupted, a dentin-healing process is initiated [22] which involves activation of pulp stem cells and pulp tissue regeneration [23]. Consequently, pulp stem cells have been used to test molecules that are to be used in restorative dentistry [24]. In addition to the biological properties described previously, another ideal characteristic of an endodontic/restorative material is its capacity to induce biomineralization. Previous studies showed that BD treatment resulted in the odontogenic differentiation of DPSC [10]. However, in order to characterize the biomineralization obtained, a combination of different markers has to be used to conclude that a given cell has osteoblastic or odontoblastic characteristics. In our study, the effects of the two biomaterials on the mineralization potential of human DPSC were evaluated using Alizarin Red S staining and investigated at the mRNA level after 10 days.

First, after 10 days, the cells produced mineralized nodules when in direct contact with the two biomaterials. At the mRNA level, direct contact downregulated three of the osteogenic gene markers we examined (ALP, COL1A1, OPN), while Runx2 was unmodified. These results are similar to those of Zanini et al., who detected reductions of ALP and COL1A1 [9]. We also observed upregulation of specific markers of odontoblastic expression in DPSC exposed to BD and BR. Nestin and Msx2 were increased in direct contact with BR. DSPP, which has a regulatory role in the mineralization of reparative dentin [25], was expressed by DPSC in direct contact with BD. In contrast, BR needed mineralizing conditions, i.e., phosphate ions as occurs in a dentin environment, to express DSPP. This apparent odontoblastic differentiation might explain the altered expression of type 1 collagen and fibronectin that we observed.

DPSC osteodifferentiation, proliferation and their easiness of access make these cells a good tool for new biomaterials testing. Indeed, titanium alloys or surface textures are now assessed by these cells [15,16]. Recently, they also have been studied in scaffold free tissue engineering [17]. Our study highlights another mean to study biocompatibility with DPSC: with or without direct contact. This may be useful for other biomaterials such as adhesives or pulp capping materials.

In conclusion, this new biomaterial, BR, caused a reduction in DPSC growth rate and had a very limited cytotoxic effect, with no genotoxicity. Like BD, it induced a positive cellular response, with biomineralization by direct contact, even in the absence of normal chemical cues. These results therefore suggest that BR might be applied as a pulp-capping material when a more fluid cement than BD is required. However, further *in vivo* experiments will be needed to confirm these observations.

## Supporting information

S1 Table Raw data results for the MTT assay. (XLSX)

S2 Table Primer sequences used in the study. (DOCX)

## References

[1] Smith AJ, Lesot H (2001) Induction and regulation of crown dentinogenesis: embryonic events as a template for dental tissue repair? Crit Rev Oral Biol Med 12: 425–437. 12002824 10.1177/10454411010120050501

[2] Smith AJ, Cassidy N, Perry H, Begue-Kirn C, Ruch JV, Lesot H (1995) Reactionary dentinogenesis. Int J Dev Biol 39: 273–280. 7626417

[3] Alliot-Licht B, Bluteau G, Magne D, Lopez-Cazaux S, Lieubeau B, Daculsi G, et al (2005) Dexamethasone stimulates differentiation of odontoblast-like cells in human dental pulp cultures. Cell Tissue Res 321: 391–400. doi: 10.1007/s00441-005-1115-7 15988617 10.1007/s00441-005-1115-7

[4] Gronthos S, Mankani M, Brahim J, Robey PG, Shi S (2000) Postnatal human dental pulp stem cells (DPSCs) in vitro and in vivo. Proc Natl Acad Sci U S A 97: 13625–13630. doi: 10.1073/pnas.240309797 11087820 10.1073/pnas.240309797 PMC17626

[5] Prati C, Gandolfi MG (2015) Calcium silicate bioactive cements: Biological perspectives and clinical applications. Dent Mater 31: 351–370. doi: 10.1016/j.dental.2015.01.004 25662204 10.1016/j.dental.2015.01.004

[6] Malkondu O, Karapinar Kazandag M, Kazazoglu E (2014) A review on biodentine, a contemporary dentine replacement and repair material. Biomed Res Int 2014: 160951 doi: 10.1155/2014/160951 25025034 10.1155/2014/160951 PMC4082844

[7] Camps J, Jeanneau C, El Ayachi I, Laurent P, About I (2015) Bioactivity of a Calcium Silicate-based Endodontic Cement (BioRoot RCS): Interactions with Human Periodontal Ligament Cells In Vitro. J Endod 41: 1469–1473. doi: 10.1016/j.joen.2015.04.011 26001857 10.1016/j.joen.2015.04.011

[8] Dimitrova-Nakov S, Uzunoglu E, Ardila-Osorio H, Baudry A, Richard G, Kellermann O, et al (2015) In vitro bioactivity of Bioroot RCS, via A4 mouse pulpal stem cells. Dent Mater 31: 1290–1297. doi: 10.1016/j.dental.2015.08.163 26364144 10.1016/j.dental.2015.08.163

[9] Zanini M, Sautier JM, Berdal A, Simon S (2012) Biodentine induces immortalized murine pulp cell differentiation into odontoblast-like cells and stimulates biomineralization. J Endod 38: 1220–1226. doi: 10.1016/j.joen.2012.04.018 22892739 10.1016/j.joen.2012.04.018

[10] Chang SW, Lee SY, Ann HJ, Kum KY, Kim EC (2014) Effects of calcium silicate endodontic cements on biocompatibility and mineralization-inducing potentials in human dental pulp cells. J Endod 40: 1194–1200. doi: 10.1016/j.joen.2014.01.001 25069932 10.1016/j.joen.2014.01.001

[11] Laurent P, Camps J, De Meo M, Dejou J, About I (2008) Induction of specific cell responses to a Ca(3)SiO(5)-based posterior restorative material. Dent Mater 24: 1486–1494. doi: 10.1016/j.dental.2008.02.020 18448160 10.1016/j.dental.2008.02.020

[12] Paranjpe A, Smoot T, Zhang H, Johnson JD (2011) Direct contact with mineral trioxide aggregate activates and differentiates human dental pulp cells. J Endod 37: 1691–1695. doi: 10.1016/j.joen.2011.09.012 22099907 10.1016/j.joen.2011.09.012 PMC3223385

[13] Gandolfi MG, Pagani S, Perut F, Ciapetti G, Baldini N, Mongiorgi R, et al (2008) Innovative silicate-based cements for endodontics: a study of osteoblast-like cell response. J Biomed Mater Res A 87: 477–486. doi: 10.1002/jbm.a.31795 18186045 10.1002/jbm.a.31795

[14] Fan C, Wang DA (2015) Effects of permeability and living space on cell fate and neo-tissue development in hydrogel-based scaffolds: a study with cartilaginous model. Macromol Biosci 15: 535–545. doi: 10.1002/mabi.201400453 25557976 10.1002/mabi.201400453

[15] Mangano C, De Rosa A, Desiderio V, d'Aquino R, Piattelli A, De Francesco F, et al (2010) The osteoblastic differentiation of dental pulp stem cells and bone formation on different titanium surface textures. Biomaterials 31: 3543–3551. doi: 10.1016/j.biomaterials.2010.01.056 20122719 10.1016/j.biomaterials.2010.01.056

[16] Naddeo P, Laino L, La Noce M, Piattelli A, De Rosa A, Iezzi G, et al (2015) Surface biocompatibility of differently textured titanium implants with mesenchymal stem cells. Dent Mater 31: 235–243. doi: 10.1016/j.dental.2014.12.015 25582059 10.1016/j.dental.2014.12.015

[17] Paino F, La Noce M, Giuliani A, De Rosa A, Mazzoni S, Laino L, et al (2017) Human DPSCs fabricate vascularized woven bone tissue: a new tool in bone tissue engineering. Clin Sci (Lond) 131: 699–713. 28209631 10.1042/CS20170047 PMC5383003

[18] Nikolova T, Dvorak M, Jung F, Adam I, Kramer E, Gerhold-Ay A, et al (2014) The gammaH2AX assay for genotoxic and nongenotoxic agents: comparison of H2AX phosphorylation with cell death response. Toxicol Sci 140: 103–117. doi: 10.1093/toxsci/kfu066 24743697 10.1093/toxsci/kfu066

[19] Luo Z, Li D, Kohli MR, Yu Q, Kim S, He WX (2014) Effect of Biodentine on the proliferation, migration and adhesion of human dental pulp stem cells. J Dent 42: 490–497. doi: 10.1016/j.jdent.2013.12.011 24440605 10.1016/j.jdent.2013.12.011

[20] Okabe T, Sakamoto M, Takeuchi H, Matsushima K (2006) Effects of pH on mineralization ability of human dental pulp cells. J Endod 32: 198–201. doi: 10.1016/j.joen.2005.10.041 16500225 10.1016/j.joen.2005.10.041

[21] Nowicka A, Lipski M, Parafiniuk M, Sporniak-Tutak K, Lichota D, Kosierkiewicz A, et al (2013) Response of human dental pulp capped with biodentine and mineral trioxide aggregate. J Endod 39: 743–747. doi: 10.1016/j.joen.2013.01.005 23683272 10.1016/j.joen.2013.01.005

[22] Stanley HR, Pereira JC, Spiegel E, Broom C, Schultz M (1983) The detection and prevalence of reactive and physiologic sclerotic dentin, reparative dentin and dead tracts beneath various types of dental lesions according to tooth surface and age. J Oral Pathol 12: 257–289. 6193259 10.1111/j.1600-0714.1983.tb00338.x

[23] Simon SR, Berdal A, Cooper PR, Lumley PJ, Tomson PL, Smith AJ (2011) Dentin-pulp complex regeneration: from lab to clinic. Adv Dent Res 23: 340–345. doi: 10.1177/0022034511405327 21677089 10.1177/0022034511405327

[24] Soares DG, Basso FG, Hebling J, de Souza Costa CA (2015) Immediate and late analysis of dental pulp stem cells viability after indirect exposition to alternative in-office bleaching strategies. Clin Oral Investig 19: 1013–1020. doi: 10.1007/s00784-014-1321-3 25248948 10.1007/s00784-014-1321-3

[25] Chen S, Gluhak-Heinrich J, Wang YH, Wu YM, Chuang HH, Chen L, et al (2009) Runx2, osx, and dspp in tooth development. J Dent Res 88: 904–909. doi: 10.1177/0022034509342873 19783797 10.1177/0022034509342873 PMC3045537

