# Supplementary material for: In vitro effects of two silicate-based materials, Biodentine and BioRoot RCS, on dental pulp stem cells in models of reactionary and reparative dentinogenesis
Source: PLoS One. 2018 Jan 25;13(1):e0190014. doi: 10.1371/journal.pone.0190014 (PMC5784909; doi:10.1371/journal.pone.0190014)
Supplement: S2 Table — (DOCX) [file pone.0190014.s002.docx]

**S 2 Table. Primer sequences used in the study. (Supplemental Data)**

| **Gene** | **Primer sequence (5’>3’)** | **Amplicon size (bp)** | **Accession number** |
| --- | --- | --- | --- |
| *SDHA* | F:AGC AAG CTC TAT GGA GAC CT  R:TAA TCG TAC TCA TCA ATC CG | 200 | NM_004168.3 |
| *UBC* | F:GTG GCA CAG CTA GTT CCG T  R:CTT CAC GAA GAT CTG CAT TGT CA | 98 | NM_021009 |
| *COL1A1* | F:AAC CAA GGC TGC AAC CTG GA  R:GGC TGA GTA GGG TAC ACG CAG G | 60 | NM_000088.3 |
| *RUNX2* | F:AAC CCA CGA ATG CAC TAT CCA  R:CGG ACA TAC CGA GGG ACA TG | 76 | NM_001024630 |
| *DSPP* | F:GCA GTG ATG AAT CTA ATG GC  R:CTG ATT TGC TGC TGT CTG AC | 489 | NM_014208.3 |
| *ALPL* | F: CGT GGC TAA GAA TGT CAT CATGTT  R: GAT TTC CCA GCG TCC TTG GC | 332 | NM_000478.4 |
| *NESTIN* | F: CAGCGTTGGAACAGAGGTTGG  R: GGCTGGCACAGGTGTCTCAAG | 392 | NM_006617.1 |
| *OPN* | F: AGC CAG GAC TCC ATT GAC TCG AAC  R: GTT TCA GCA CTC TGG TCA TCC AGC | 416 | NM_001040058.1 |
| *DLX5* | F: GACTTCCAAGCTCCGTTCCA  R: GGCAAAGTTGGCGATTCCTG | 70 | NM_005221 |
| *MSX2* | F: GCACCCTGAGGAAACACAAGA  R: CGAGGAGCTGGGATGTGGTA | 67 | NM_002449 |
| *MMP9* | F: CAC TAC TGT GCC TTT GAG TCC  R: CGA TGG CGT CGA AGA TGT T | 62 | NM_004994 |
